# Identifying subgroups with decreased performance on malaria rapid diagnostic tests in Malawi

**DOI:** 10.1371/journal.pgph.0007366

**Published:** 2026-11-03

**Authors:** Geoffrey Guenther, Alick Sixpence, Charles Mangani, Alfred Matengeni, Jaroslaw Harezlak, Don P. Mathanga, Terrie E. Taylor, Miriam K. Laufer, Karl B. Seydel, Lauren M. Cohee, Clarissa Valim

**Affiliations:** 1 Division of Infectious Diseases, Department of Pediatrics, Boston Children’s Hospital, Boston, Massachusetts, United States of America; 2 Margaret C. Ryan Global Health Program, Boston Children’s Hospital, Boston, Massachusetts, United States of America; 3 Department of Pediatrics, Harvard Medical School, Boston, Massachusetts, United States of America; 4 Department of Epidemiology, Boston University School of Public Health, Boston, Massachusetts, United States of America; 5 MAC Communicable Diseases Alert Centre, Kamuzu University of Health Sciences, Blantyre, Malawi; 6 Department of Community and Environmental Health, School of Global and Public Health, Kamuzu University of Health Sciences, Blantyre, Malawi; 7 Department of Epidemiology and Biostatistics, Indiana University School of Public Health, Bloomington, Indiana, United States of America; 8 Department of Osteopathic Medical Specialties, Michigan State University College of Osteopathic Medicine, East Lansing, Michigan, United States of America; 9 Blantyre Malaria Project, Kamuzu University of Health Sciences, Blantyre, Malawi; 10 Center for Vaccine Development and Global Health, University of Maryland School of Medicine, Baltimore, Maryland, United States of America; 11 Department of Clinical Sciences, Liverpool School of Tropical Medicine, Liverpool, United Kingdom; 12 Department of Global Health, Boston University School of Public Health, Boston, Massachusetts, United States of America; Tulane University School of Public Health and Tropical Medicine, UNITED STATES OF AMERICA

## Abstract

We sought to identify groups in which performance of point-of-care malaria rapid diagnostic tests (MRDTs) was decreased to improve the accuracy of MRDT-based diagnosis. We evaluated the performance of *Plasmodium falciparum* MRDTs compared to quantitative polymerase chain reaction (PCR) in population subgroups based on 1108 patient visits to rural health centers in Malawi from 2019 to 2021. Approximately 48% of malaria PCRs and 37% of MRDTs were positive. Overall, the fraction of positive MRDTs among participants with positive PCR (sensitivity) was 67% and the fraction of negative MRDTs among participants with negative PCR (specificity) was 91%. Sensitivity varied with age, lowest among subjects aged 25–40 years (38%) and < 1 year (33%) and highest among those 5–8 years (86%). Nearly all cases of MRDT-negative/PCR-positive discordance (false negative MRDTs) had < 200 parasites/µl of blood. In regression models, male sex, younger age, and presence of fever, headache, chills, and vomiting reduced the likelihood of a false negative result. Presence of fever, headache, chills, and vomiting were more likely to result in a false positive result. Using classification tree analysis, combinations of clinical and demographic characteristics predicted true positive and true negative MRDT results with 79% and 84% overall accuracy, respectively. Age and parasite density were negatively correlated; age was included in regression models and classification trees rather than parasite density because it would be information readily available to clinicians. Though there are several possible explanations for discordance between PCR and MRDT (including incidental malaria infections), MRDTs performed less well in certain subgroups, which may inform deployment and development of these important tools in clinical settings and for malaria surveillance.

## Introduction

Malaria is the parasitic disease with the largest impact on global health, with an estimated 282 million cases resulting in more than 600,000 deaths in 2024, mostly in children aged under 5 years [1]. One of the main approaches recommended by the World Health Organization (WHO) to control the burden of malaria is the rapid diagnosis and treatment of all suspected cases. Effectiveness of this approach depends on accurate diagnosis. *Plasmodium falciparum* malaria rapid diagnostic tests (MRDTs) are cost-effective tools that extend access to testing to the point-of-care where skilled microscopy generally cannot be performed [2]. The most common MRDTs are based on the detection of histidine-rich protein 2 (HRP2), while others detect *Plasmodium* lactate dehydrogenase (pLDH) or aldolase [3,4]. The WHO standard is that MRDTs should detect infection down to a diagnostic threshold of 200 parasites/μL of blood [5], whereas microscopy has a lower limit of detection of approximately 100 parasites/μL [2]. In a 2011 systematic review of real-world MRDT performance in uncomplicated malaria compared to microscopy, the average sensitivity and specificity of HRP2-based MRDTs were 95% although sensitivity varied across studies from 42% to 100% and specificity ranged from 65% to 100%; pLDH-based tests were only slightly less sensitive and more specific on average [6].

Recent data on MRDT performance suggest a more nuanced picture. In several studies, MRDT sensitivity and specificity varied significantly with age and parasite density. Sensitivity appeared to decrease as patients became older, possibly due to lower parasite density resulting from repeated exposure and increased immunity over time [7,8]. Compared to PCR, conventional HRP2-based MRDTs only detected about 50% of infections at parasite densities less than 100/μL and only about 70% of infections at parasite densities between 1,000 and 10,000/μL in a study in Mozambique [8]. In a study in Ethiopia, pLDH-based MRDTs had a sensitivity of 69% when compared to PCR [9]. The emergence of HRP2 deletion may partially explain decreased sensitivity in some regions [10]. Though there is marked heterogeneity in the prevalence of this deletion [11] it has recently been reported at significant rates in northern and central Malawi [12]. Further, cases missed by MRDT may represent subclinical malaria infections, in which symptoms are attributable to other causes and not malaria.

Decreased specificity may be partially explained by the slow clearance of HRP2, which can persist for several weeks after successful antimalarial treatment [13], though how much this phenomenon contributes to false positive cases is unknown. In the same Ethiopian study mentioned above, pLDH-based MRDTs had a specificity of 38% when compared to PCR [9]. Specificity was as low as 25% in young children aged 5–9 years in a study in Tanzania [14].

Although MRDTs continue to be essential for case management of malaria, it would be important in different malaria-endemic settings to identify subgroups in whom MRDT performance is reduced and their associated parasite density and infection history. Because MRDTs may miss infections, combining MRDTs with clinical signatures obtained through algorithms implementable at point-of-care can help improve accuracy and distinguish clinical from subclinical or incidental malaria infections. Recently, researchers have used machine learning to develop predictive models for malaria diagnosis using clinical and demographic features, though these features differed between low- and high-intensity transmission areas [15]. Here, comparing presence of parasites on quantitative PCR versus MRDT result, we sought to identify population subgroups in which performance of MRDTs was decreased and approaches to improve the accuracy of MRDT-based diagnosis in an area of moderate-intensity transmission.

## Materials and methods

### Ethics statement

The study received ethical approval from the National Health Science Review Committee in Malawi and the Institutional Review Boards of Michigan State University, the University of Maryland School of Medicine, Boston University, and Boston Children’s Hospital. Written informed consent was obtained from the head of each household and from all adults living in the household. For each child under 18 years, the consent of one parent or guardian was also obtained. Assent was obtained from children aged 13–17 years.

### Study procedures

Participants were enrolled in a community-based cohort study from the catchment areas of two rural health centers in southern Malawi: Ntaja (Machinga District) and Namanolo (Balaka District). These catchment areas were each divided into 4 quadrants in which 4 index households per quadrant were sampled. All members of the index household plus the surrounding 4–6 closest neighboring households were invited to join the study during the follow-up period. Participants were instructed to report to their respective health center when they experienced any illness. At these passive case detection [PCD] visits, participants or their guardians were interviewed to record symptoms, duration, and prior treatment for the presenting illness. When clinically indicated by the health care provider, an MRDT was performed and a dried blood sample was collected. DNA was extracted using the methanol method [16], and the *Plasmodium falciparum* 18S ribosomal RNA gene was detected and quantified using qPCR [17]. Based on government provided stocks, brands of MRDTs in use varied slightly over time and by site (S1 Table), though all were HRP2-based and the most common tests (Paracheck Pf Device, Orchid Biomedical Systems, India; SD Bioline Malaria Ag P.f, Abbott Diagnostics, Republic of Korea) remained consistent over time and site. Recruitment and enrollment occurred from April 2, 2019 to March 20, 2020, though PCD visits continued through October 7, 2021.

### Definitions

False negative results were defined as a negative MRDT given a positive PCR, and false positive results were defined as a positive MRDT given a negative PCR. These terms were used for clarity, but do not imply the presence or absence of clinically meaningful malaria infection; parasite PCR is not an optimal gold standard to establish a diagnosis of clinical malaria, since parasitemia may coexist with other pathogens that might be the real drivers of the clinical disease. In analyses, fever was defined as either a measured temperature of ≥37.5°C at the PCD visit or reported fever during the illness.

### Statistical analysis

Visits for which either MRDT or PCR results were missing were excluded from all analyses and summary statistics (216 out of 1324 visits, 16%). We compared demographic and clinical variables between participants with positive vs. negative MRDTs and PCRs using Wilcoxon tests for continuous and ordinal variables, and chi-square or Fisher exact tests for categorical variables. Operational characteristics of MRDTs were estimated through sensitivity (and false negative fraction), specificity (and false positive fraction), and positive and negative predictive values (PPV and NPV) with corresponding 95% confidence intervals. To investigate demographic and clinical characteristics that would result in higher odds of false positive or negative results of the MRDTs, we utilized logistic generalized additive mixed models, with random intercepts to account for correlation of repeated PCD visits of each participant. Analyses were conducted separately for PCR-positive and PCR-negative subsets of the data using MRDT results as outcomes. In these models, we assessed parasite density and other participant characteristics: sex, age at visit, education level of the head of household, socioeconomic status score, whether the visit occurred during the rainy (November-April) or dry (May-October) season, whether the participant lived in a finished house, had had a PCD visit in the preceding 30 days with or without a positive MRDT, and whether the participant reported any of the following symptoms during the illness: fever, chills, muscle pain, headache, fatigue, vomiting, diarrhea, anorexia, or cough. Characteristics were selected for final models if their coefficients were statistically significant (p-value < 0.05) or if their inclusion impacted the coefficient of other variables in the model. To better accommodate nonlinearity in associations between age and parasite density with false positive and negative results, we added these terms to models using penalized splines.

Using classification tree models, we explored combinations of predictors of true negative and positive MRDT results. We excluded parasite density from these models, since this information would not be readily available to clinicians at the point-of-care. In the analysis of both PCR-positive and PCR-negative visits, we applied loss matrices to penalize misclassification of a true positive and a true negative MRDT, respectively. PPV and NPV for the classification trees were determined from sensitivity, specificity, and overall malaria positivity as a surrogate for prevalence using standard equations [18].

All analyses were performed using R Statistical Software version 4.4.1 (R Foundation for Statistical Computing, Vienna, Austria) using the mgcv (v1.9-1), rpart (v4.1.24), ggplot2 (v3.5.1), gratia (v0.10.0), and plot.rpart (v3.1.2) packages.

## Results

Between 2019 and 2021, 1108 PCD visits were completed among 468 participants from 174 households (Table 1), with an average of 2.4 PCD visits per participant (range of 1–20 visits). Approximately 48% of malaria PCR tests were positive, compared to 37% of MRDTs. As expected, the likelihood of a positive MRDT was higher during the high transmission rainy season (62%) than the dry, low transmission season (33%). Participants or their caregivers reported having previously sought care for the presenting illness at 5% of PCD visits. Though response rates were low (~23%) for follow-up questions about specific types of treatment they had received during the illness, only 1% reported having taken antimalarials.

**Table 1:** Participant characteristics at PCD visits by test result.

	All (N = 1108)	MRDT (–)(N = 697)	MRDT (+)(N = 411)	P value (+ v. – MRDT)^a^	PCR (–)(N = 573)	PCR (+)(N = 535)	P value (+ v. – PCR)^a^
**Demographics**							
**Age at visit (years),** median [IQR]	13.0 [5.9, 26.4]	16.9 [5.2, 28.8]	10.5 [6.2, 15.9]	< 0.0001	16.9 [4.9, 28.6]	10.1 [6.1, 18.6]	< 0.0001
**Male** (%)	341 (31%)	178 (26%)	163 (40%)	< 0.0001	145 (25%)	196 (37%)	< 0.0001
**Site**							
A (%)	332 (30%)	228 (33%)	104 (25%)	0.009	193 (34%)	139 (26%)	0.005
B (%)	776 (70%)	469 (67%)	307 (75%)	–	380 (66%)	396 (74%)	–
**Socioeconomic status score** ^**b**^ **, median [IQR]**	8.98 [5.2, 14.5]	8.98 [5.3, 14.4]	8.98 [4.9, 15.2]	0.48	8.96 [5.6, 13.2]	8.99 [4.9, 16.4]	0.74
**Socioeconomic status tertile** ^**c**^							
Lowest (%)	253 (24%)	150 (23%)	103 (26%)	0.30	122 (23%)	131 (26%)	0.62
Middle (%)	649 (62%)	406 (63%)	243 (62%)	–	335 (63%)	314 (61%)	–
Highest (%)	140 (13%)	94 (15%)	46 (12%)	–	73 (14%)	67 (13%)	–
**Household head education level** ^**†**^							
None (%)	246 (27%)	144 (25%)	1021(29%)	0.16	108 (24%)	138 (30%)	0.14
Any primary school (%)	386 (42%)	234 (41%)	152 (43%)	–	196 (43%)	190 (41%)	–
Secondary school or beyond (%)	289 (31%)	191 (34%)	98 (28%)	–	150 (33%)	139 (30%)	–
**Visit occurred during rainy season** (%)	621 (56%)	369 (53%)	252 (62%)	0.007	289 (50%)	332 (62%)	< 0.0001
**Participant lives in a finished house**^**†**^ (%)	384 (37%)	255 (39%)	129 (33%)	0.04	205 (39%)	179 (35%)	0.21
**Laboratory Results**							
**Positive malaria PCR** (%)	535 (48%)	174 (25%)	361 (88%)	< 0.0001	–	–	–
**Positive MRDT** (%)	411 (37%)	–	–	–	50 (9%)	361 (68%)	< 0.0001
**History / Physical Examination**							
**Objective fever (T ≥ 37.5°C)** (%)	234 (21%)	64 (9%)	170 (41%)	< 0.0001	57 (10%)	177 (33%)	< 0.0001
**Duration of illness prior to presentation (days),** median [IQR]	2.0 [2,3]	2.0 [2, 3]	2.0 [2, 3]	0.91	2.0 [2, 3]	2.0 [2, 3]	0.90
**Previously sought treatment for this illness (%)**	54 (5%)	41 (6%)	13 (3%)	0.04	34 (6%)	20 (4%)	0.10
**Reported symptoms during illness:**							
**Fever** (%)	669 (61%)	363 (52%)	306 (75%)	< 0.0001	313 (55%)	356 (67%)	< 0.0001
**Chills** (%)	237 (22%)	87 (13%)	150 (37%)	< 0.0001	82 (14%)	155 (29%)	< 0.0001
**Muscle pain** (%)	380 (34%)	233 (34%)	147 (36%)	0.39	199 (35%)	181 (34%)	0.79
**Headache** (%)	524 (47%)	283 (41%)	241 (59%)	< 0.0001	247 (43%)	277 (52%)	0.003
**Fatigue** (%)	41 (4%)	29 (4%)	12 (3%)	0.30	24 (4%)	17 (3%)	0.38
**Vomiting** (%)	167 (15%)	54 (8%)	113 (28%)	< 0.0001	52 (9%)	115 (22%)	< 0.0001
**Diarrhea** (%)	90 (8%)	73 (11%)	17 (4%)	0.0002	57 (10%)	33 (6%)	0.02
**Anorexia** (%)	19 (2%)	10 (1%)	9 (2%)	0.34	11 (2%)	8 (2%)	0.59
**Cough**^**†**^ (%)	266 (29%)	205 (38%)	61 (16%)	< 0.0001	156 (36%)	110 (24%)	< 0.0001

Comparison of characteristics between PCD visits with positive and negative malaria rapid diagnostic test (MRDT) results, and with positive and negative malaria polymerase chain reaction (PCR) tests.

^a^P-values calculated via Wilcoxon rank-sum tests for continuous and ordinal variables, and chi-square or Fisher exact tests for categorical variables.

^b^Calculated using inverse probability weights of socioeconomic indicators including possession of a radio, television, mobile phone, bicycle, knives, chairs, tables, bed, mattress, cabinet, livestock, and private toilet

^c^Percentages are calculated among valid responses. For socioeconomic status, household head education level, finished house, and cough variables, there were only 1042, 921, 1042, and 909 valid responses, respectively.

The most commonly reported symptoms at PCD visits were fever and headache, and, as expected, participants with positive MRDTs were more likely to report those symptoms (75% vs 52% for fever, 59% vs 41% for headache). Those with positive MRDTs were also more likely to report vomiting (26% vs 8%), and chills (37% vs 13%) but less likely to report diarrhea (4% vs 11%,) or cough (16% vs 38%). All these differences were statistically significant (p-value < 0.05) (Table 1).

Overall, when compared to PCR, 174 of 697 negative MRDT results (25%) were false negative (MRDT-negative given PCR-positive), and 50 of 411 positive MRDT results (12%) were false positive (MRDT-positive given PCR-negative).

### Identifying subgroups at higher risk of false positive and negative MRDT results

As expected, parasite density exhibited the strongest effect on performance of the MRDT (Fig 1). False negative MRDT results mostly occurred in participants with parasite density less than 100 parasites/μL. Only 11 (6%) false negative results had a parasite density above the standard MRDT diagnostic threshold of 200 parasites/μL, all in participants 3–18 years of age (median 11 years, IQR 9–14), with a median parasite density of 2,146 parasites/µl (IQR 1,192–11,501). High-density false negative results were more likely in the dry season, whereas false negatives in the study as a whole were more common in the rainy season. All high-density false negative results occurred in one site, and most (8/11) occurred in 2021, toward the end of the study period (S2 Table).

**Fig 1:**
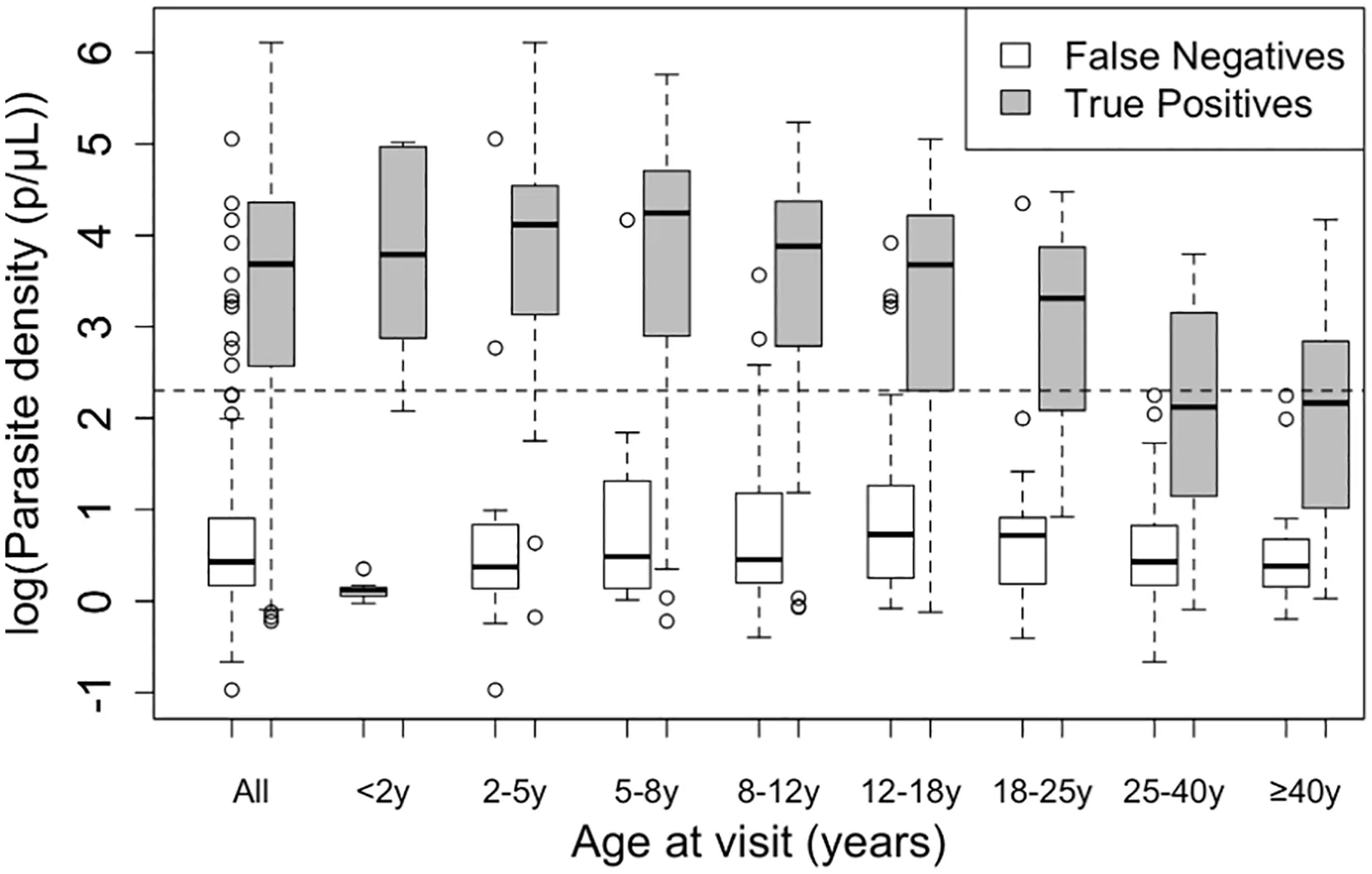
Parasite density by age subgroup at PCD visits. Log-transformed parasite density in PCR-positive tests by age subgroup at PCD visit. Dashed horizontal line represents the WHO-established diagnostic threshold for MRDTs of 200 parasites/µL, also log-transformed.

MRDT sensitivity (67%), a direct function of false negative results, varied substantially by age at the time of PCD visit, peaking at 5–8 years (86%) and 8–12 years (83%) (Fig 2), and was lower at the extremes of age. Sensitivity in children under five was low (63%), though specificity was high at 93%. Notably, sensitivity was particularly poor in infants under 1 year (2 false negative / 3 positive; sensitivity 33%), though there were only seven visits in this age group. Sensitivity decreased after age 18 (50% in 18–25 years, 38% in 25–40 years). PPV correlated with sensitivity in terms of age-based performance. Specificity was high at 91% overall and varied less with age.

**Fig 2:**
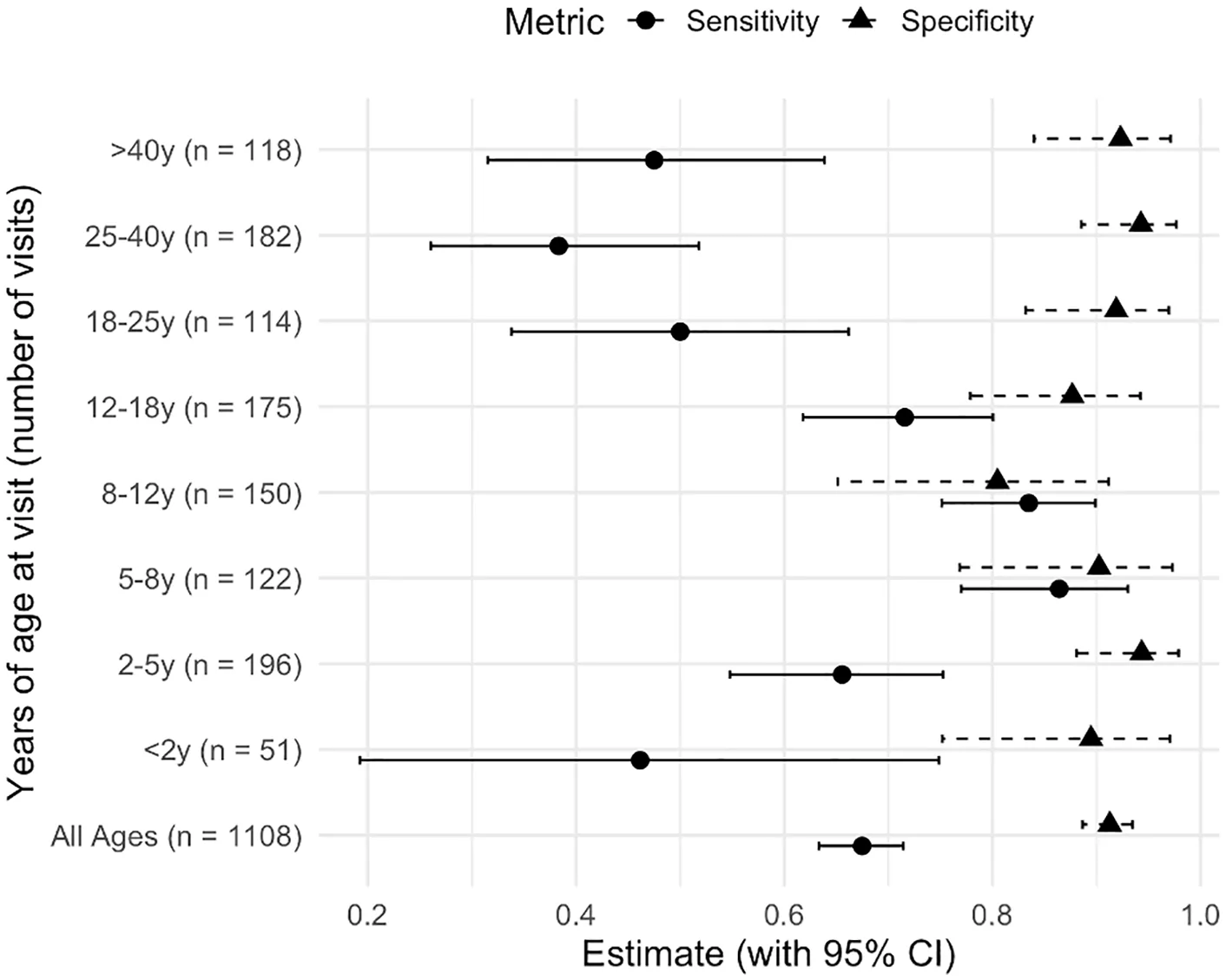
MRDT performance characteristics by age subgroup at PCD visits.

Age trends in false negative MRDT fraction were also present in crude regression analysis: the probability of false negative results was high in the youngest participants and decreased with age until approximately 8 years of age, when it started increasing (Fig 3A). After adjusting for demographic and clinical variables, younger participants had lower odds of false negative results and the association with age was approximately linear (Fig 3B). In addition, female participants and participants with fever, chills, headache, and vomiting had reduced odds of false negative results (Table 2). The dramatic decline in the predicted probability of false negative results in the first few years of life in crude analysis was not seen in the adjusted model, as the youngest participants had very low probability of reporting chills, headache, and vomiting, factors associated with a lower probability of false negatives. Age at visit and log-transformed parasite density were negatively correlated, consistent with the observed decline in parasite density with increasing age. We chose to include age in final models given that age, unlike parasite density, is a clinical variable available to the clinician at the point of care. Additionally, model fitness as suggested by the Akaike Information Criteria (AIC), was better in the model including age (2563.3) than in the model including parasite density (3325.7). Notably, associations with clinical characteristics in models including age and parasite density were comparable (S3 Table).

**Table 2:** The effect of predictors in final models for false negative and false positive MRDT results.

False Negative		
**Predictor**	**Odds Ratio**	**95% Confidence Interval**
Male sex	0.54	(0.32, 0.89)
Fever (measured or reported)	0.29	(0.17, 0.48)
Headache	0.26	(0.16, 0.40)
Chills	0.23	(0.13, 0.40)
Vomiting	0.16	(0.08, 0.30)
Age at visit	Varies^a^	Varies^a^
**False Positive**		
**Predictor**	**Odds Ratio**	**95% Confidence Interval**
Vomiting	2.60	(1.14, 5.90)
Chills	2.31	(1.14, 4.69)
Fever (measured or reported)	2.17	(1.10, 4.27)
Headache	1.99	(1.07, 3.69)

^a^Age was included as a continuous predictor in a non-linear model (see Fig 3b); predicted probability of a false negative result increased with rising age

**Fig 3:**
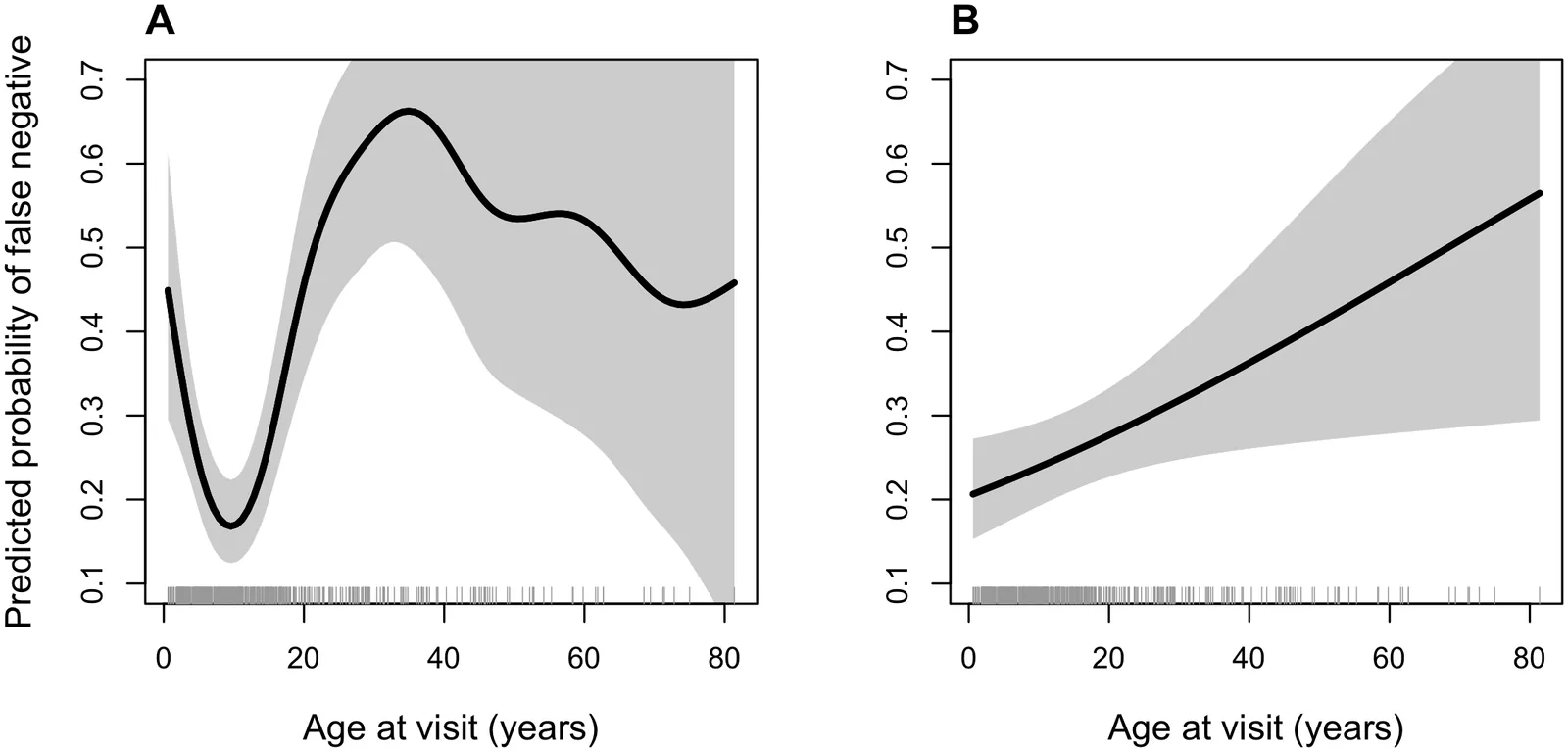
Effect of age on operational characteristics of MRDTs. In (A), the crude effect of age on probability of false negative MRDT result for PCR-positive visits is shown, with the corresponding 95% confidence intervals (shaded area). In (B), the effect of age on probability of false negative MRDT result for PCR-positive visits is adjusted for other covariates: sex, fever, headache, chills, and vomiting. Covariates were held at their mean values. Vertical lines at the bottom of the figures represent data points at the specified age.

Age was not a statistically significant predictor of false positive results. Among those with negative PCR, participants with fever, chills, headache, and vomiting had increased odds of false positive MRDT results in the adjusted model (Table 2). Notably, no participants with false positive results had a preceding PCD visit with a positive MRDT within 30 days prior. Only 2 participants with false positive MRDT results reported having sought care for the presenting illness prior to the PCD visit; both reported prior use of antimalarial medications.

### Algorithm to predict true positive and negative MRDT results

Classification trees of the PCR-positive subset were split based on a number of clinical and demographic factors, including age and symptoms (Fig 4). For example, a participant with fever, under the age of 18, without headache but with chills would be predicted to have a true positive result. This tree achieved a sensitivity of 80% and a specificity of 77% for the detection of true positive MRDT results, and an overall accuracy of 79%.

**Fig 4:**
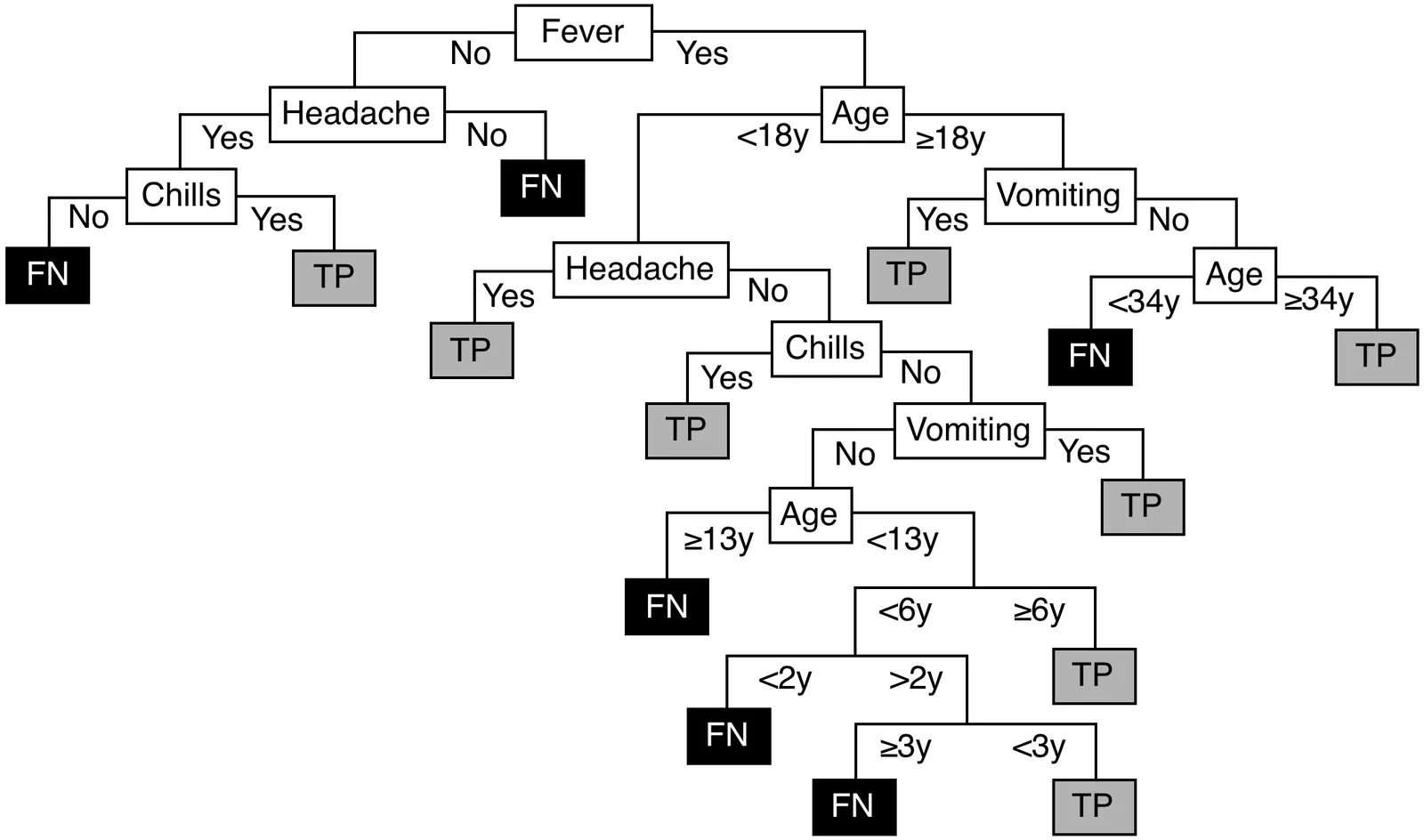
Signatures predicting a true positive MRDT result obtained through classification trees. Among PCR-positive PCD visits. Terminal nodes represent predicted true positives (TP, gray) or false negative (FN, black). The signature attained a sensitivity of 0.80 and specificity of 0.77 (overall accuracy of 0.79).

The classification tree for the PCR-negative subset included many of the same clinical and demographic features as the PCR-positive subset, as well as whether the visit occurred during the rainy season, and whether the participant lived in a finished house (Fig 5). This classification tree achieved a sensitivity of 85% and a specificity of 80% for the detection of true negative MRDT results, and an overall accuracy of 84%.

**Fig 5:**
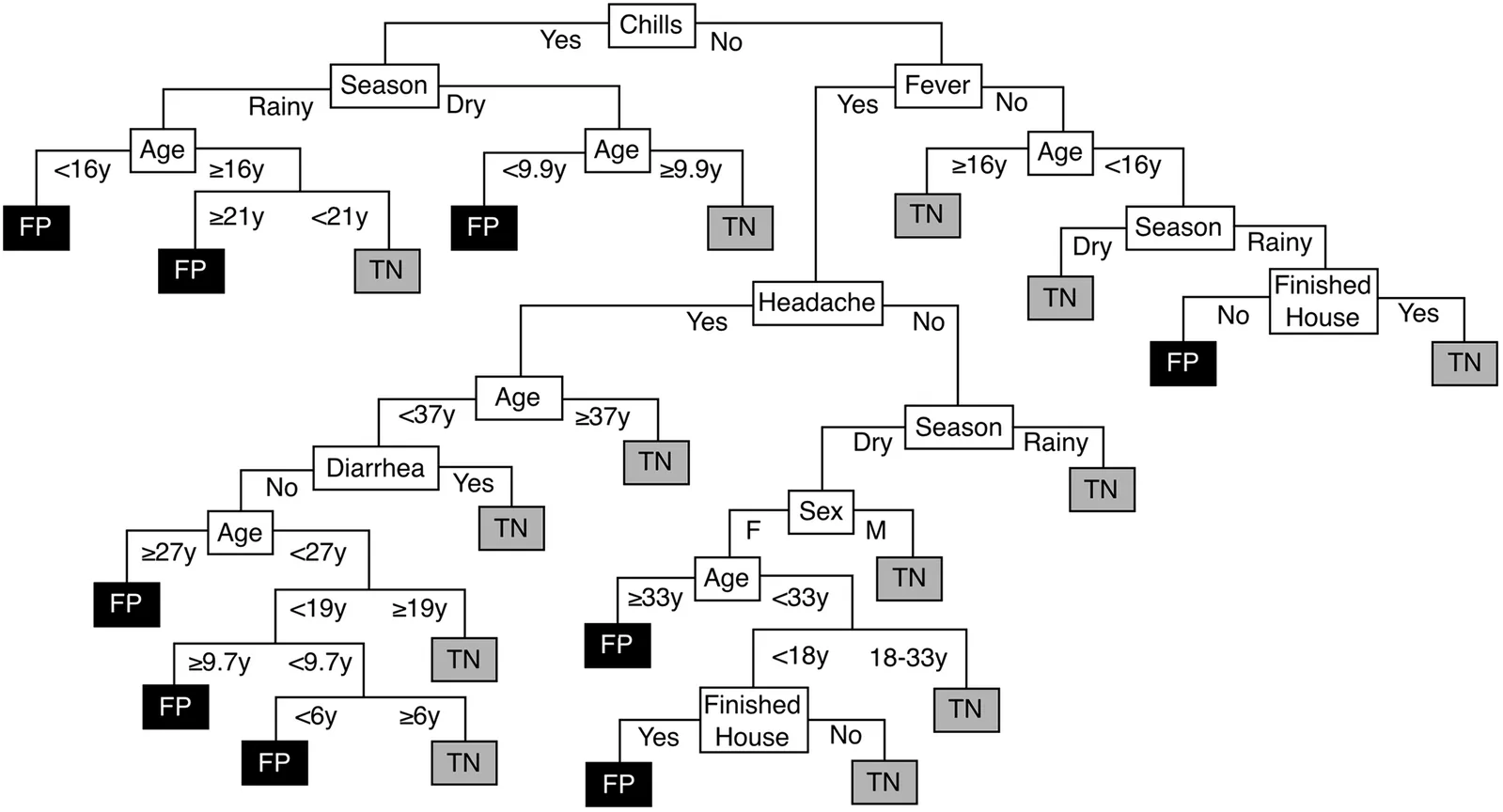
Signatures predicting a true negative MRDT result obtained through classification trees. Among PCR-negative PCD visits. Terminal nodes represent predicted true negatives (TN, gray) or false positives (FP, black). The signature attained a sensitivity of 0.85, specificity of 0.80 (overall accuracy of 0.84).

When using these classification tree algorithms, the clinician should consider the local prevalence of malaria. In our study, with a malaria test positivity rate at health center visits (surrogate measure for prevalence) of 48%, the trees demonstrated a PPV of 77% for the PCR-positive tree and an NPV of 85% for the PCR-negative tree. These predictive values will change with increasing or decreasing prevalence. For example, with a prevalence of 60%, the PPV increases to 84% and the NPV decreases to 73%.

## Discussion

Overall, operational characteristics of MRDT were within previously published ranges [6]. However, as in other studies, MRDT performance was variable. Generally, suspected malaria infections confirmed by PCR and missed by MRDTs had low parasite densities, although MRDTs detected some infections with parasite density below the 200 parasites/μL threshold and missed some above this threshold.

Future studies are needed to better understand the clinical significance of very-low-density malaria parasitemia. Negative MRDT with positive PCR results in patients with parasite density below the MRDT diagnostic threshold could be explained by incidental parasitemia and an alternate etiology responsible for symptoms (e.g., viral infection). Indeed, the odds of a false negative result were higher in participants without common malaria symptoms including fever, chills, headache, and vomiting. Because study sites were in rural, resource-constrained healthcare settings there were no other diagnostic tests available such as X-ray or viral testing. Therefore, our ability to establish an alternative cause of febrile illness for the false negative MRDT cases was limited.

Alternatively, a portion of negative MRDT results with positive PCR could have represented true clinical malaria with parasite densities below the MRDT diagnostic threshold. Prior studies suggest that the pyrogenic threshold, the minimum level of parasitemia associated with a febrile episode, is decreased in younger children [19–21] and in areas with lower transmission rates [21–23]. Some studies have found a pyrogenic threshold less than 200 parasites/μL in young children and infants [20,24]. Submicroscopic malaria infections, those detected by molecular techniques but not by microscopy, have been associated with higher risk of fever in studies in children in Rwanda and Uganda [25,26]. An earlier Malawian study found that submicroscopic infection was associated with an increase in the odds of fever in the rainy season, though this was not statistically significant; interestingly, during the dry season such infections were associated with a statistically significant decrease in the odds of fever [27].

The 11 high-density false negative cases all occurred in one site and mostly toward the end of the study period, which together raise the possibility that HRP2 deletion may have contributed to the MRDT negative results. HRP2 deletion was not known to be present in Malawi at the time of the study, so HRP2 genotyping was not performed. One study conducted at the same time as ours found high rates (10–15%) of this deletion in the Central and Northern regions of Malawi [12], though currently there does not appear to be evidence of high rates of this deletion in the Southern region where our study was conducted. The fact that, in our study, low-density false negative cases were far more common than high-density cases may point to low parasite density as the main driver of false negative MRDTs. The clustering in time and location may also point to operator or device error as contributors, though we could not ascertain either the test operators or specific brand of MRDT for each individual encounter.

Like other studies [7,8,14], we found that MRDT performance was highly variable with age, with sensitivity dropping off precipitously after age 18, and with median parasite density for true positives below the diagnostic threshold in the oldest subgroups (>25 years). Also concerning, although sample size was limited, were the findings of high rates of false negative results in children under five years old, who are at substantial risk of severe malaria-related morbidity and mortality – a risk that is not always well correlated with parasite density [28–30]. Our results prompt the question of whether a more sensitive diagnostic modality should be considered for people at the extremes of age and in areas or seasons of low malaria transmission who are at highest risk of low-level parasitemia that could be missed by MRDTs. This is particularly true for national malaria surveillance programs and research studies of malaria epidemiology using MRDTs.

False positive results (i.e., positive MRDT with negative PCR) were more likely in those presenting with fever, chills, headache, or vomiting. The underlying reason for false positives remains unexplained. As noted above, no visits with false positive results were preceded by a PCD visit with a positive MRDT within 30 days, and only 2 were preceded by reported use of antimalarial medication. These findings may make it less likely that large numbers of false positive results were due to lingering HRP2 antigen from a prior treated malaria infection, though lack of detailed information on treatment history limits the conclusions that can be drawn. False positive results may lead to suboptimal patient management and increased burden on health systems with unnecessary use of antimalarial treatment or the inappropriate withholding of indicated antibiotic therapy. False positive malaria infection results may also challenge malaria surveillance efforts.

This study has many strengths, most notably the large sample size encompassing various age groups, and the extended follow-up period. There are some limitations as well. MRDTs detect parasite proteins while PCR detects parasite DNA, which both differ from microscopy which detects whole parasites. This may affect direct comparisons between the testing modalities. The target of the MRDT (HRP2) differed from that of the PCR. We did not test for HRP2 deletion that could have resulted in false negative results. Additionally, there was variation in the MRDT brands used across the study sites and time, and our PCR testing focused exclusively on detection of *P. falciparum*; recent surveillance in Malawi shows a prevalence of *P. malariae* of 10–15% among those with parasitemia, particularly in asymptomatic cases [31–33]. Finally, although our overall sample size was substantial, it was not sufficient to reliably partition the data into independent training and validation sets for the clinical diagnosis algorithms proposed here.

Future studies with more comprehensive clinical characterization of patients are necessary to validate our findings. Following patients after diagnosis and treatment could help determine whether MRDT misclassification affects clinical outcomes. Additional research could also evaluate the feasibility and cost-effectiveness of deploying more sensitive diagnostic modalities in the age groups with higher rates of false positive and negative results. A clinical diagnosis algorithm could be incorporated in a mobile phone application and support diagnosis at the point-of-care. This could make malaria diagnosis less dependent on parasite density thresholds and result in more effective management for vulnerable populations.

## Supporting information

S1 Table Brands of MRDT used during the study. (DOCX)

S2 Table Characteristics of high-density false negative cases (MRDT-negative / PCR-positive). (DOCX)

S3 Table Comparison between the age-based and density-based logistic regression models for false negative results. Because age at visit and log-transformed parasite density were correlated and thus could not be included in the same model, we conducted a separate analysis to assess whether the effects of sex and symptoms on the odds of a false-negative MRDT result were consistent when age was substituted for parasite density. All other model terms other than parasite density were held identical. The direction of effect for fever, vomiting, headache, and chills was consistent across both models, with each symptom associated with reduced odds of a false-negative result regardless of whether age or parasite density was used as the covariate. Effect magnitudes were somewhat attenuated in the density-based model. The association with male sex was the only one that differed in the two models: male sex was significantly associated with higher odds of false-negative results in the age-based model (p = 0.017) but was not statistically significant in the parasite density-based model (p = 0.521). The age-based model showed substantially better fit than the density-based model (Akaike Information Criteria [AIC] = 2563.3 vs. 3325.7). Predicted probabilities of a false-negative result from the two models were positively correlated (Spearman’s ρ = 0.73), indicating broadly consistent classification of the risk of false negative MRDT results across participants despite the difference in model fit and the divergent finding for sex.
(DOCX)

## References

[1] World Health Organization. World Malaria Report 2025. Geneva: World Health Organization. 2024.

[2] World Health Organization. WHO Guidelines for Malaria. Geneva: World Health Organization; 2023.

[3] Gatton ML, Dunn J, Chaudhry A, Ciketic S, Cunningham J, Cheng Q. Implications of parasites lacking Plasmodium falciparum histidine-rich protein 2 on malaria morbidity and control when rapid diagnostic tests are used for diagnosis. J Infect Dis. 2017;215(7):1156–66. doi: 10.1093/infdis/jix094 28329034

[4] Chiodini PL, Bowers K, Jorgensen P, Barnwell JW, Grady KK, Luchavez J, et al. The heat stability of Plasmodium lactate dehydrogenase-based and histidine-rich protein 2-based malaria rapid diagnostic tests. Trans R Soc Trop Med Hyg. 2007;101(4):331–7. doi: 10.1016/j.trstmh.2006.09.007 17212967

[5] World Health Organization. Malaria Rapid Diagnostic Test Performance: Results of WHO Product Testing of Malaria RDTs: Round 8 (2016–2018). Geneva: World Health Organization. 2018.

[6] Abba K, Deeks JJ, Olliaro P, Naing C-M, Jackson SM, Takwoingi Y, et al. Rapid diagnostic tests for diagnosing uncomplicated P. falciparum malaria in endemic countries. Cochrane Database Syst Rev. 2011;2011(7):CD008122. doi: 10.1002/14651858.CD008122.pub2 21735422 PMC6532563

[7] Chinkhumba J, Skarbinski J, Chilima B, Campbell C, Ewing V, San Joaquin M, et al. Comparative field performance and adherence to test results of four malaria rapid diagnostic tests among febrile patients more than five years of age in Blantyre, Malawi. Malar J. 2010;9:209. doi: 10.1186/1475-2875-9-209 20646312 PMC2916916

[8] Galatas B, Mayor A, Gupta H, Balanza N, Jang IK, Nhamussua L, et al. Field performance of ultrasensitive and conventional malaria rapid diagnostic tests in southern Mozambique. Malar J. 2020;19(1):451. doi: 10.1186/s12936-020-03526-9 33287822 PMC7720469

[9] Feleke SM, Gidey B, Mohammed H, Nega D, Dillu D, Haile M, et al. Field performance of Plasmodium falciparum lactate dehydrogenase rapid diagnostic tests during a large histidine-rich protein 2 deletion survey in Ethiopia. Malar J. 2022;21(1):236. doi: 10.1186/s12936-022-04257-9 35971118 PMC9377056

[10] Molina-de la Fuente I, Pastor A, Herrador Z, Benito A, Berzosa P. Impact of Plasmodium falciparum pfhrp2 and pfhrp3 gene deletions on malaria control worldwide: a systematic review and meta-analysis. Malar J. 2021;20(1):276. doi: 10.1186/s12936-021-03812-0 34158065 PMC8220794

[11] World Health Organization Global Malaria Programme. Malaria Threats Map: Parasite pfhrp2/3 Gene Deletions. 2025. https://apps.who.int/malaria/maps/threats/ Accessed 2025 January 28.

[12] Louis JM, Mazigo E, Jun H, Lee W-J, Syahada JH, Fitriana F, et al. First report of pfhrp2 and pfhrp3 gene deletions compromising HRP2-based malaria rapid diagnostic tests in Malawi. Infect Dis Poverty. 2025;14(1):98. doi: 10.1186/s40249-025-01368-8 41068992 PMC12512526

[13] Poti KE, Sullivan DJ, Dondorp AM, Woodrow CJ. HRP2: Transforming Malaria Diagnosis, but with Caveats. Trends Parasitol. 2020;36(2):112–26. doi: 10.1016/j.pt.2019.12.004 31848119

[14] Laurent A, Schellenberg J, Shirima K, Ketende SC, Alonso PL, Mshinda H, et al. Performance of HRP-2 based rapid diagnostic test for malaria and its variation with age in an area of intense malaria transmission in southern Tanzania. Malar J. 2010;9:294. doi: 10.1186/1475-2875-9-294 20974009 PMC2974751

[15] Mariki M, Mkoba E, Mduma N. Combining clinical symptoms and patient features for malaria diagnosis: machine learning approach. Appl Artif Intell. 2022;36(1). doi: 10.1080/08839514.2022.2031826

[16] Bereczky S, Mårtensson A, Gil JP, Färnert A. Short report: Rapid DNA extraction from archive blood spots on filter paper for genotyping of Plasmodium falciparum. Am J Trop Med Hyg. 2005;72(3):249–51. doi: 10.4269/ajtmh.2005.72.249 15772315

[17] Hermsen CC, Telgt DS, Linders EH, van de Locht LA, Eling WM, Mensink EJ, et al. Detection of Plasmodium falciparum malaria parasites in vivo by real-time quantitative PCR. Mol Biochem Parasitol. 2001;118(2):247–51. doi: 10.1016/s0166-6851(01)00379-6 11738714

[18] Altman DG, Bland JM. Diagnostic tests 2: Predictive values. BMJ. 1994;309(6947):102. doi: 10.1136/bmj.309.6947.102 8038641 PMC2540558

[19] Rogier C, Commenges D, Trape JF. Evidence for an age-dependent pyrogenic threshold of Plasmodium falciparum parasitemia in highly endemic populations. Am J Trop Med Hyg. 1996;54(6):613–9. doi: 10.4269/ajtmh.1996.54.613 8686780

[20] McGuinness D, Koram K, Bennett S, Wagner G, Nkrumah F, Riley E. Clinical case definitions for malaria: clinical malaria associated with very low parasite densities in African infants. Trans R Soc Trop Med Hyg. 1998;92(5):527–31. doi: 10.1016/s0035-9203(98)90902-6 9861370

[21] Mwangi TW, Ross A, Snow RW, Marsh K. Case definitions of clinical malaria under different transmission conditions in Kilifi District, Kenya. J Infect Dis. 2005;191(11):1932–9. doi: 10.1086/430006 15871128 PMC3545188

[22] Dicko A, Mantel C, Kouriba B, Sagara I, Thera MA, Doumbia S, et al. Season, fever prevalence and pyrogenic threshold for malaria disease definition in an endemic area of Mali. Trop Med Int Health. 2005;10(6):550–6. doi: 10.1111/j.1365-3156.2005.01418.x 15941418

[23] Dollat M, Talla C, Sokhna C, Diene Sarr F, Trape J-F, Richard V. Measuring malaria morbidity in an area of seasonal transmission: Pyrogenic parasitemia thresholds based on a 20-year follow-up study. PLoS One. 2019;14(6):e0217903. doi: 10.1371/journal.pone.0217903 31246965 PMC6597048

[24] Roucher C, Rogier C, Dieye-Ba F, Sokhna C, Tall A, Trape J-F. Changing malaria epidemiology and diagnostic criteria for Plasmodium falciparum clinical malaria. PLoS One. 2012;7(9):e46188. doi: 10.1371/journal.pone.0046188 23029433 PMC3460864

[25] Sifft KC, Geus D, Mukampunga C, Mugisha JC, Habarugira F, Fraundorfer K, et al. Asymptomatic only at first sight: malaria infection among schoolchildren in highland Rwanda. Malar J. 2016;15(1):553. doi: 10.1186/s12936-016-1606-x 27842542 PMC5109732

[26] Katrak S, Nayebare P, Rek J, Arinaitwe E, Nankabirwa JI, Kamya M, et al. Clinical consequences of submicroscopic malaria parasitaemia in Uganda. Malar J. 2018;17(1):67. doi: 10.1186/s12936-018-2221-9 29402282 PMC5800031

[27] Vareta J, Buchwald AG, Barrall A, Cohee LM, Walldorf JA, Coalson JE, et al. Submicroscopic malaria infection is not associated with fever in cross-sectional studies in Malawi. Malar J. 2020;19(1):233. doi: 10.1186/s12936-020-03296-4 32600362 PMC7322713

[28] von Seidlein L, Olaosebikan R, Hendriksen ICE, Lee SJ, Adedoyin OT, Agbenyega T, et al. Predicting the clinical outcome of severe falciparum malaria in African children: findings from a large randomized trial. Clin Infect Dis. 2012;54(8):1080–90. doi: 10.1093/cid/cis034 22412067 PMC3309889

[29] Gonçalves BP, Huang C-Y, Morrison R, Holte S, Kabyemela E, Prevots DR, et al. Parasite burden and severity of malaria in Tanzanian children. N Engl J Med. 2014;370(19):1799–808. doi: 10.1056/NEJMoa1303944 24806160 PMC4091983

[30] Olupot-Olupot P, Engoru C, Nteziyaremye J, Chebet M, Ssenyondo T, Muhindo R, et al. The clinical spectrum of severe childhood malaria in Eastern Uganda. Malar J. 2020;19(1):322. doi: 10.1186/s12936-020-03390-7 32883291 PMC7470679

[31] Gumbo A, Topazian HM, Mwanza A, Mitchell CL, Puerto-Meredith S, Njiko R, et al. Occurrence and Distribution of Nonfalciparum Malaria Parasite Species Among Adolescents and Adults in Malawi. J Infect Dis. 2022;225(2):257–68. doi: 10.1093/infdis/jiab353 34244739 PMC8763954

[32] Community Health Services Unit NMCP. National Malaria Strategic Plan (2023-2030). Lilongwe: Malawi Ministry of Health. 2023.

[33] Bowa C, Kalonga N, Mbonshi B, Sitali L, Simwela J, Kudowa E, et al. Prevalence and Plasmodium species distribution of asymptomatic malaria parasitemia among blood donors at the Lilongwe blood transfusion center. Front Malar. 2025;3:1687355. doi: 10.3389/fmala.2025.1687355

