# Supplementary material for: Identifying subgroups with decreased performance on malaria rapid diagnostic tests in Malawi
Source: PLOS Glob Public Health. 2026 Nov 3;6(11):e0007366. doi: 10.1371/journal.pgph.0007366 (PMC13730336; doi:10.1371/journal.pgph.0007366)
Supplement: Supplementary file 1 [file pgph.0007366.s001.docx]

**S1 Table.**

| Site / Year | Primary MRDT(s) | Alternative MRDT |
| --- | --- | --- |
| **Namanolo** |  |  |
| 2019 | Paracheck Pf Device^a^ | SD Bioline Malaria Ag P.f^b^ |
| 2020 | Paracheck Pf Device^a^ | SD Bioline Malaria Ag P.f^b^ |
| 2021 | Paracheck Pf Device^a^ | SD Bioline Malaria Ag P.f^b^ |
| **Ntaja** |  |  |
| 2019 | SD Bioline Malaria Ag P.f^b^  Paracheck Pf Device^a^ | STANDARD Q Malaria P.f Ag Test^c^ |
| 2020 | Paracheck Pf Device^a^  SD Bioline Malaria Ag P.f^b^  First Response Malaria Ag *P. falciparum* (HRP2) Card Test^d^ | ParaHIT - f Device^e^ |
| 2021 | SD Bioline Malaria Ag P.f^b^  Paracheck Pf Device^a^  ParaHIT - f Device^e^ | None |

^a^Orchid Biomedical Systems, India

^b^Abbott Diagnostics, Republic of Korea

^c^SD Biosensor, Republic of Korea

^d^Premier Medical Corporation, India

^e^Arkray Healthcare, India
