# Supplementary material for: Identifying subgroups with decreased performance on malaria rapid diagnostic tests in Malawi
Source: PLOS Glob Public Health. 2026 Nov 3;6(11):e0007366. doi: 10.1371/journal.pgph.0007366 (PMC13730336; doi:10.1371/journal.pgph.0007366)
Supplement: Supplementary file 2 [file pgph.0007366.s002.docx]

**S2 Table.**

| **Age at visit (years)** | **Year of visit** | **Sex** | **Season** | **Site** | **qPCR Parasite Density (p/µL)** | **Fever** | **Chills** | **Muscle Pain** | **Headache** | **Fatigue** | **Vomiting** | **Diarrhea** | **Anorexia** |
| --- | --- | --- | --- | --- | --- | --- | --- | --- | --- | --- | --- | --- | --- |
| 3 | 2021 | F | Rainy | B | 113702 | Yes | No | No | No | No | No | Yes | No |
| 4 | 2021 | F | Rainy | B | 587 | Yes | No | Yes | No | No | No | No | No |
| 7 | 2021 | F | Dry | B | 14716 | No | No | No | No | No | No | Yes | No |
| 10 | 2021 | F | Rainy | B | 382 | Yes | No | Yes | Yes | No | No | No | No |
| 10 | 2019 | F | Dry | B | 738 | Yes | Yes | No | Yes | No | No | No | No |
| 11 | 2021 | F | Dry | B | 3683 | Yes | No | No | Yes | No | Yes | No | No |
| 12 | 2021 | F | Dry | B | 8286 | Yes | No | No | Yes | No | No | No | No |
| 13 | 2021 | M | Dry | B | 1893 | Yes | No | No | No | No | No | Yes | No |
| 14 | 2021 | F | Dry | B | 2146 | No | No | No | Yes | No | No | No | No |
| 15 | 2020 | M | Rainy | B | 1647 | Yes | No | No | Yes | No | No | No | No |
| 18 | 2020 | F | Dry | B | 22309 | Yes | Yes | Yes | Yes | No | No | No | No |
