# Supplementary material for: Identifying subgroups with decreased performance on malaria rapid diagnostic tests in Malawi
Source: PLOS Glob Public Health. 2026 Nov 3;6(11):e0007366. doi: 10.1371/journal.pgph.0007366 (PMC13730336; doi:10.1371/journal.pgph.0007366)
Supplement: Supplementary file 3 [file pgph.0007366.s003.docx]

**S3 Table.**

| **Predictor** | **Model including age** | | **Model including parasite density** | |
| --- | --- | --- | --- | --- |
|  | Odds Ratio | 95% Confidence Interval | Odds Ratio | 95% Confidence Interval |
| Male sex | 0.54 | (0.32, 0.89) | 0.81 | (0.43, 1.54) |
| Fever (measured or reported) | 0.29 | (0.17, 0.48) | 0.51 | (0.27, 0.93) |
| Headache | 0.26 | (0.16, 0.40) | 0.37 | (0.21, 0.68) |
| Chills | 0.23 | (0.13, 0.40) | 0.35 | (0.17, 0.70) |
| Vomiting | 0.16 | (0.08, 0.30) | 0.33 | (0.13, 0.81) |
